# Single-center clinical analysis of hybrid aortic arch repair for aortic arch lesions

**DOI:** 10.3389/fcvm.2026.1797260

**Published:** 2026-04-21

**Authors:** Yang Gao, Yilingrui Wang, Quanhua Pan, Chuanzhang Xu, Lijuan Zhang, Zhiwei Xu

**Affiliations:** Department of Cardiothoracic Surgery, The Affiliated Huai’an No.1 People’s Hospital of Nanjing Medical University, Huai’an, Jiangsu, China

**Keywords:** aortic arch lesions, efficacy, hybrid aortic arch repair, safety, single-center study

## Abstract

**Objective:**

To evaluate the safety and short-to-mid-term efficacy of hybrid aortic arch repair (HAR) within a structured, anatomy- and risk-based decision-making framework for heterogeneous aortic arch lesions.

**Methods:**

Forty-five consecutive patients with aortic arch lesions who underwent HAR between January 2019 and January 2024 were retrospectively included. Lesions comprised degenerative arch aneurysm (46.7%), non-A non-B aortic dissection (33.3%), residual arch disease after Type A repair (13.3%), and penetrating ulcers (6.7%). Preoperative computed tomography angiography and EuroSCORE II were used for anatomical assessment and risk stratification. Based on a two-dimensional principle of anatomical suitability and comorbidity risk, patients were assigned to Type I HAR (*n* = 2), Type II HAR (*n* = 16), or Type IV HAR (*n* = 27). Type I involved debranching with staged Zone 0 thoracic endovascular aortic repair (TEVAR), Type II consisted of total arch replacement with stented elephant trunk followed by TEVAR, and Type IV used cervical bypass with one-stage Zone 2 TEVAR. All decisions were confirmed by a multidisciplinary team.

**Results:**

All procedures were technically successful, with no intraoperative or 30-day mortality. Type I HAR showed the shortest operative time and least blood loss, whereas Type II had the highest surgical complexity and longest recovery. Postoperative complications were minor, and no stroke, renal failure, or permanent organ injury occurred. During a median follow-up of 18 months (range, 6–36 months), no aortic-related death, endoleak, reintervention, or branch vessel occlusion occurred. Aorta-related survival and freedom from reintervention were both 100%.

**Conclusion:**

Individualized HAR guided by anatomical features and patient risk provides a safe and effective treatment for complex aortic arch lesions, particularly in high-risk patients unsuitable for open surgery.

## Introduction

Aortic arch diseases encompass a heterogeneous group of life-threatening conditions, including aortic arch aneurysms, aortic dissections involving the arch (Stanford Type A and non-A non-B dissections), congenital arch malformations, and penetrating aortic ulcers (1). Among these entities, aortic arch aneurysms and acute Type A aortic dissections represent the most critical clinical scenarios. Previous epidemiological studies have demonstrated that the rate of mortality of untreated acute Type A aortic dissection increases by approximately 1%–2% per hour during the first 48 h, and the 30-day mortality rate exceeds 50% without timely surgical intervention (2). Likewise, rupture of an aortic arch aneurysm is associated with an extremely high early mortality rate, reported to be greater than 80%, underscoring the urgent need for effective therapeutic strategies (3, 4).

Conventional open aortic arch replacement remains the gold standard for definitive treatment; however, it requires cardiopulmonary bypass (CPB) and deep hypothermic circulatory arrest (DHCA). These procedures are associated with substantial surgical trauma, massive blood loss, and challenges in cerebral protection, with reported perioperative stroke rates ranging from 4% to 12% (5). Moreover, open arch surgery is frequently complicated by multiorgan dysfunction, including acute kidney injury and respiratory failure. In elderly patients or those with significant comorbidities such as hypertension, diabetes, and chronic obstructive pulmonary disease (COPD), perioperative risk is markedly increased, with 30-day mortality rates reported to exceed 20% (6, 7). Consequently, a considerable proportion of patients are deemed unsuitable for conventional open repair.

Thoracic endovascular aortic repair (TEVAR) offers a less invasive alternative and avoids thoracotomy and CPB, thereby reducing perioperative morbidity. However, its application is limited in aortic arch disease because an adequate proximal landing zone (≥2–3 cm) is often unavailable when lesions involve supra-aortic branches. This anatomical constraint predisposes to endoleak, stent migration, and cerebral ischemic events, making isolated TEVAR unsuitable for complex arch pathology (8).

To overcome these limitations, hybrid aortic arch repair (HAR) has been developed, integrating supra-aortic debranching with endovascular stent grafting. This approach preserves cerebral perfusion while creating a secure proximal landing zone, thereby combining the durability of open surgery with the minimally invasive advantages of endovascular techniques (9).

Given the intrinsic heterogeneity of aortic arch pathology and the absence of universally accepted modality selection criteria, a structured decision-making approach may be more clinically relevant than evaluating any single technique in isolation. Therefore, the objective of this study was not merely to report clinical outcomes of HAR but to evaluate whether an individualized, anatomy- and risk-based modality selection strategy could achieve favorable short- to mid-term outcomes in patients with heterogeneous aortic arch lesions, particularly in high-risk populations who are unsuitable for conventional open repair.

## Materials and methods

### Ethics

This study was conducted in accordance with the Declaration of Helsinki and was approved by the Ethics Committee of The Affiliated Huai’an No. 1 People’s Hospital of Nanjing Medical University. Informed consent was not applicable due to the retrospective nature of this study.

### Study subjects

A total of 45 consecutive patients with aortic arch lesions who underwent HAR at our institution between January 2019 and January 2024 were retrospectively included. The cohort comprised 29 men and 16 women, with a mean age of 62.3 ± 8.5 years (range, 48–76 years). Lesion types included degenerative aortic arch aneurysm in 21 patients (46.7%), non-A non-B aortic dissection in 15 (33.3%), residual arch lesions after Type A aortic dissection repair in 6 (13.3%), and penetrating aortic ulcers in 3 (6.7%). Major comorbidities were hypertension in 41 patients (91.1%), hyperlipidemia in 28 (62.2%), coronary artery disease in eight (17.8%), and COPD in five (11.1%). None of the patients had a history of previous sternotomy.

### Classification and definition of HAR modalities

In the present study, HAR was defined as a treatment strategy combining open supra-aortic vessel reconstruction with endovascular stent graft implantation, regardless of whether cardiopulmonary bypass was required in selected complex cases. Based on institutional practice and anatomical characteristics, HAR was categorized into three types:

1. *Type I HAR*: indicated for patients with localized proximal arch lesions and a normal ascending aorta (diameter <4 cm). This strategy consisted of supra-aortic debranching via a small upper sternal incision followed by staged Zone 0 TEVAR, without DHCA.
2. *Type II HAR*: applied to patients with pan-arch disease requiring ascending aorta and total arch reconstruction. This modality included total arch replacement combined with stented elephant trunk implantation (Stage I), followed by staged TEVAR to the descending aorta (Stage II). CPB with moderate hypothermia (28°C–30°C) was used for cerebral protection.
3. *Type IV HAR*: indicated for patients with distal arch lesions extending into the proximal descending aorta and severe cardiopulmonary dysfunction (e.g., EF < 40%, FEV1 < 1.5 L). This approach involved left common carotid artery–left subclavian artery bypass via a small left cervical incision, followed by one-stage Zone 2 TEVAR, without sternotomy or CPB.

### Surgical modality selection process

Modality selection followed a three-step process:

1. *Anatomical assessment*: Preoperative coronary artery CT (CTA) was used to measure key parameters: (1) aortic arch diameter (diameter >4 cm indicates high risk of ascending aortic lesions); (2) proximal anchoring zone length (Zone 0 < 2 cm requires anchoring zone extension); (3) branch vessel involvement (whether the orifices of the brachiocephalic trunk and left common carotid artery are complicated with plaques/dissections); and (4) lesions at the origin of the descending aorta (diameter >4.5 cm or dissection involvement requires coverage).
2. *Risk stratification*: The European System for Cardiac Operative Risk Evaluation II (EuroSCORE II) was used for risk assessment: (1) low risk (≤4 points): Tolerant of sternotomy, priority given to evaluating eligibility for Type Ⅰ HAR; (2) high risk (>4 points): With severe comorbidities (e.g., chronic heart failure and chronic lung disease), evaluating eligibility for Type Ⅳ HAR; and (3) very high risk (>8 points): Only Type Ⅳ HAR (no CPB or sternotomy) is considered.
3. *Modality matching*: (1) “Normal ascending aorta + anchoring zone ≥2 cm + low risk” → Type Ⅰ HAR; (2) “Pan-arch lesions + need for ascending aortic replacement + low/moderate risk” → Type Ⅱ HAR; and (3) “Distal arch lesions + high/very high risk + inability to tolerate sternotomy” → Type Ⅳ HAR.

**Figure 1:**
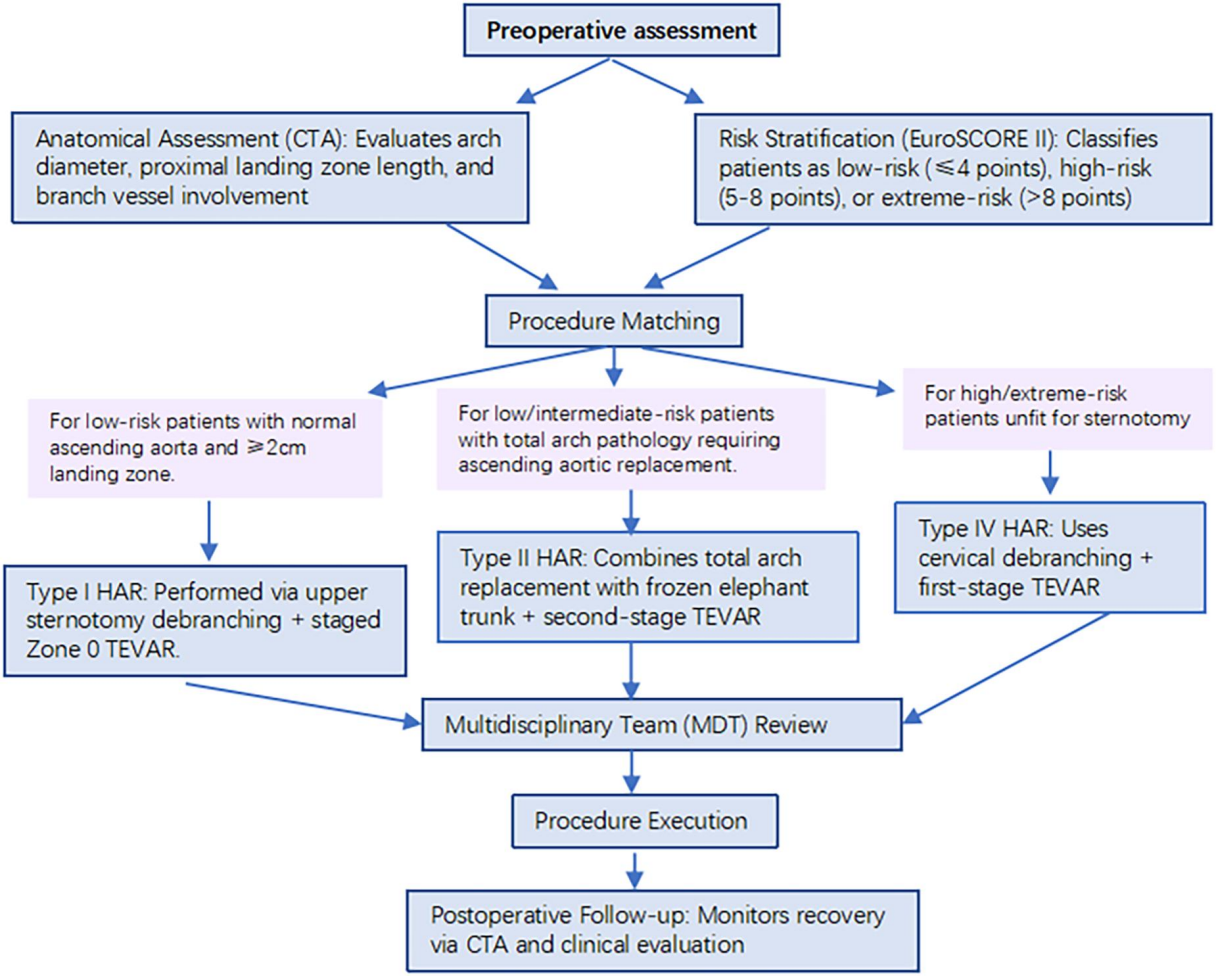
Flowchart of surgical modality selection. Preoperative assessment: anatomical assessment (CTA: arch diameter, anchoring zone length, branch lesions); Risk stratification (EuroSCORE Ⅱ: low risk ≤4 points, high risk 5–8 points, very high risk >8 points); Modality matching: Low risk + normal ascending aorta + anchoring zone ≥2 cm → Type Ⅰ HAR (upper sternal debranching + staged Zone 0 TEVAR); low/moderate risk + pan-arch lesions + need for ascending aortic replacement → Type Ⅱ HAR (total arch replacement + stented elephant trunk + staged TEVAR); High/very high risk + inability to tolerate sternotomy → Type Ⅳ HAR (cervical debranching + Zone 2 TEVAR); MDT consultation confirmation; Surgery implementation; Postoperative follow-up: CTA + clinical assessment.

All modality selections were confirmed by multidisciplinary team (MDT) consultation involving cardiac surgery, vascular surgery, and radiology departments (Figure 1).

Patients with proximal aortic arch lesions who were assessed as high or prohibitive surgical risk were not considered suitable candidates for hybrid repair in our institutional protocol. In these cases, conventional elephant trunk repair under cardiopulmonary bypass was preferred, as it allowed more controlled proximal reconstruction and cerebral protection. Such patients were therefore not included in the present hybrid cohort.

### Key surgical technical points

1. *Type I HAR (two cases)*: In Stage I, a 5 cm small upper sternal incision was made. After dissecting the ascending aorta, a partial occlusion clamp was applied, and an 8 mm Dacron branched graft was implanted (one end anastomosed to the anterior wall of the ascending aorta, and the other end anastomosed to the right common carotid artery and left common carotid artery, respectively). In Stage II (3–5 days postoperatively), Zone 0 TEVAR was performed via femoral artery puncture, with the proximal stent covering the ascending aortic graft to ensure no endoleak.
2. *Type II HAR (16 cases)*: In Stage I, under general anesthesia, a median sternotomy was performed, and CPB was established (aortic root perfusion+superior vena cava drainage). The diseased total arch was resected under moderate hypothermia (28°C), and a total arch graft with SET was implanted (the distal SET was inserted into the descending aorta for 2–3 cm). Brachiocephalic vessels were anastomosed to the graft branches. In Stage II (2 weeks postoperatively), TEVAR was performed via femoral artery, with the proximal stent overlapping the SET by ≥3 cm.
3. *Type IV HAR (27 cases)*: Under general anesthesia, a 4 cm small left cervical incision was made. The left common carotid artery and left subclavian artery were dissected, and a 6 mm polytetrafluoroethylene bypass graft was implanted. Immediately after surgery, Zone 2 TEVAR was performed via right femoral artery puncture: the proximal stent was located 1 cm distal to the left common carotid artery orifice, and the distal stent covered descending aortic lesions without covering the left subclavian artery.

### Postoperative follow-up and efficacy evaluation

1. *Short-term assessment (1 month postoperatively)*: CTA reexamination to evaluate stent position and endoleak; detection of liver/kidney function and troponin (to rule out myocardial injury).
2. *Medium-term assessment (6–12 months postoperatively)*: CTA reexamination to evaluate aortic diameter changes (presence of dilatation); echocardiography to assess left ventricular function.
3. *Long-term assessment (annually)*: Clinical follow-up to record symptoms (chest pain, hoarseness); CTA to monitor stent stability and branch vessel patency.
4. *Efficacy indicator definitions*: (1) Technical success: accurate stent placement, no Type Ⅰ/Ⅲ endoleak; and (2) Clinical success: No death, stroke, or permanent complications; patients resume normal activities.

## Results

### Baseline data and modality distribution

Among the 45 patients, there were two patients with Type Ⅰ HAR (low risk, normal ascending aorta + small arch aneurysm), 16 with Type Ⅱ HAR (low/moderate risk, pan-arch aneurysm + ascending aortic dilatation), and 27 with Type Ⅳ HAR (high/very high risk, 22 patients with chronic heart failure/COPD). Complete baseline data were available for all patients and each modality group, covering different lesion types and comorbidities, providing a comprehensive basis for efficacy analysis (Table 1).

**Table 1:** Baseline data and modality distribution of patients.

Indicator	Type I HAR (*n* = 2)	Type II HAR (*n* = 16)	Type IV HAR (*n* = 27)	Total (*n* = 45)
Age (years)	58.5 ± 7.2	61.8 ± 8.9	63.2 ± 8.1	62.3 ± 8.5
Gender (*n*, %)				
Male	1 (50.0)	11 (68.8)	17 (63.0)	29 (64.4)
Female	1 (50.0)	5 (31.2)	10 (37.0)	16 (35.6)
Lesion type (*n*, %)				
Aortic arch aneurysm	1 (50.0)	8 (50.0)	12 (44.4)	21 (46.7)
Type B dissection	1 (50.0)	5 (31.2)	9 (33.3)	15 (33.3)
Residual Type A dissection	0 (0.0)	3 (18.8)	3 (11.1)	6 (13.3)
Comorbidities (*n*, %)				
Hypertension	2 (100.0)	15 (93.8)	24 (88.9)	41 (91.1)
Hyperlipidemia	1 (50.0)	10 (62.5)	17 (63.0)	28 (62.2)
Coronary heart disease	0 (0.0)	3 (18.8)	5 (18.5)	8 (17.8)
COPD	0 (0.0)	2 (12.5)	3 (11.1)	5 (11.1)
EuroSCORE II (points)	3.0 ± 0.0	4.5 ± 1.2	9.2 ± 2.1	7.1 ± 3.2

HAR, hybrid aortic arch repair; COPD, chronic obstructive pulmonary disease.

### Surgery and early postoperative efficacy

All patients completed surgery successfully, with no intraoperative deaths or 30-day mortality. Type Ⅰ HAR (small upper sternal incision) involved the simplest operation, shortest operation time, and least intraoperative blood loss. Type Ⅱ HAR (requiring total arch replacement and CPB) had the highest complexity, longest operation time, maximum blood loss, and a relatively long postoperative hospital stay. Type Ⅳ HAR (minimally invasive small cervical incision) had operation time and blood loss between Type Ⅰ and Type Ⅱ HAR. Postoperative complications were all minor in nature; no severe adverse events (stroke, acute renal failure) occurred. All patients had their endotracheal tubes removed within 72 h postoperatively, with smooth recovery (Table 2).

**Table 2:** Surgical and early postoperative indicators of different modalities.

Indicator	Type I HAR (*n* = 2)	Type II HAR (*n* = 16)	Type IV HAR (*n* = 27)	Total (*n* = 45)
Operation duration (min)	155.5 ± 20.3	240.2 ± 35.1	190.4 ± 25.3	198.6 ± 38.5
Intraoperative blood loss (mL)	200.2 ± 50.3	450.2 ± 80.4	250.6 ± 60.5	290.3 ± 95.7
Postoperative hospital stay (ds)	12.2 ± 1.1	15.2 ± 2.1	17.2 ± 1.3	15.8 ± 1.5
Postoperative extubation time (hs)	18.3 ± 3.2	26.0 ± 5.1	20.4 ± 4.2	22.2 ± 5.3
Minor complications (*n*, %)	0 (0.0)	1 (6.25)	2 (7.41)	3 (6.67)
Transient hoarseness	0 (0.0)	0 (0.0)	2 (7.41)	2 (4.44)
Mild upper limb swelling	0 (0.0)	1 (6.25)	0 (0.0)	1 (2.22)
Major complications (*n*, %)	0 (0.0)	0 (0.0)	0 (0.0)	0 (0.0)

HAR, hybrid aortic arch repair.

### Short-to-medium-term follow-up results

All 45 patients completed follow-up (100% follow-up rate), with a follow-up duration of 6–36 months (median: 18 months). Imaging evaluation showed that at 12 months postoperatively, the aortic arch diameter of patients in each modality group did not significantly dilate compared with preoperative values; stents were stable with no Type Ⅰ/Ⅲ endoleak; the patency rate of branch vessels (brachiocephalic trunk, common carotid artery) reached 100%. Clinically, no aortic-related death, stent migration, or aortic rupture occurred; both the aorta-specific survival rate and the freedom from reintervention rate were 100%. In terms of functional recovery, patients in Type Ⅰ and Type Ⅳ HAR groups returned to normal work earlier, reflecting the advantage of minimally invasive modalities in postoperative rehabilitation (Table 3).

**Table 3:** Short-to-medium-term follow-up results of the three groups.

Indicator	Type I HAR (*n* = 2)	Type II HAR (*n* = 16)	Type IV HAR (*n* = 27)	Total (*n* = 45)
Follow-up duration (months, median)	24	18	16	18
Aortic arch diameter (cm)				
Preoperative	3.8 ± 0.3	4.5 ± 0.5	4.2 ± 0.4	4.3 ± 0.5
12 months postoperatively	3.7 ± 0.2	4.3 ± 0.4	4.1 ± 0.3	4.2 ± 0.4
Patency rate of branch vessels (%)	100.0	100.0	100.0	100.0
Time to return to normal work (months)	3.0 ± 0.0	6.2 ± 1.3	3.2 ± 0.5	4.1 ± 1.8
Aorta-specific survival rate (%)	100.0	100.0	100.0	100.0
Freedom from reintervention rate (%)	100.0	100.0	100.0	100.0

HAR, hybrid aortic arch repair.

## Discussion

In this single-center cohort, individualized hybrid aortic arch repair guided by anatomical assessment and EuroSCORE II-based risk stratification achieved excellent short- to mid-term outcomes, with 100% technical success, zero perioperative mortality, no major neurological events, and complete freedom from aortic-related reintervention during follow-up. Importantly, these favorable results were observed across heterogeneous lesion types and risk categories, suggesting that appropriate modality matching—rather than advocating any single hybrid technique—may be a key determinant of clinical success in complex arch pathology. The present study should therefore be interpreted as an evaluation of a treatment allocation framework rather than a technical innovation report.

The key contribution of this study is not merely reporting favorable outcomes of HAR but demonstrating that structured modality matching may prevent both overtreatment and undertreatment in complex aortic arch disease. Excessively aggressive open arch reconstruction in fragile patients may increase perioperative mortality, whereas purely endovascular approaches in anatomically unsuitable cases may predispose to endoleak and late failure. By integrating anatomical parameters and physiological risk, our framework attempts to balance radical lesion exclusion with procedural tolerance (10). For patients with proximal arch involvement and high operative risk, conventional elephant trunk repair was favored in our center because it provides direct visualization of the proximal arch and more secure anastomosis, which was considered safer than complex hybrid configurations in this subgroup. In addition, we acknowledge that the inclusion of patients requiring cardiopulmonary bypass increases the heterogeneity of the cohort. However, our intention was not to restrict the definition of hybrid repair to procedures avoiding sternotomy or CPB, but rather to evaluate a spectrum of combined open and endovascular strategies tailored to anatomical complexity.

Previous systematic reviews have reported perioperative mortality rates of 5%–15% and stroke rates of 3%–8% for hybrid arch procedures. It is acknowledged that certain hybrid techniques, particularly cervical debranching combined with Zone 2 TEVAR for distal arch lesions, have been well established for decades. However, few studies have evaluated these modalities within a unified selection framework that simultaneously considers anatomical extent and physiological reserve. In contrast, the present cohort demonstrated zero perioperative mortality and no major neurological events (11, 12). Although direct comparison is limited by sample size and patient selection, these findings suggest that careful patient-modality matching may contribute to risk reduction beyond procedural technique alone (13–15). In patients who required left subclavian artery revascularization, cervical bypass was preferred over branched or fenestrated endovascular techniques. This decision was based on several considerations. First, a considerable proportion of our patients were relatively young, in whom long-term patency and durability were major concerns. Surgical bypass using prosthetic grafts has well-established long-term outcomes, whereas long-term durability data for branched or fenestrated arch devices remain limited. Second, device availability and procedural cost were also taken into account, as customized or branched stent grafts are associated with high economic burden. Therefore, cervical debranching was considered a more durable and pragmatic option in our institutional practice.

Several factors may explain the excellent early outcomes observed in this study. First, strict preoperative anatomical screening helped avoid taking up technically challenging cases of patients who were unsuitable for HAR. Second, the staged strategy in Type I and Type II HAR may reduce physiological burden by separating surgical trauma and endovascular contrast exposure. Third, multidisciplinary decision-making likely improved procedural appropriateness (16, 17).

From a practical standpoint, HAR should not be regarded as a universal substitute for conventional open repair. Instead, it may serve as an intermediate strategy between open arch replacement and purely endovascular repair, particularly for patients with moderate-to-high surgical risk and anatomically complex disease.

Rather than focusing on procedural nuances, the clinical implication of differentiating HAR modalities lies in matching surgical invasiveness with disease extent and physiological reserve (17). Type I HAR may be suitable for patients with localized proximal arch disease and preserved cardiopulmonary function. In the present cohort, the limited number of such cases reflects strict anatomical indications rather than selective reporting, and this modality should be interpreted as exploratory within the proposed framework. Type II HAR provides a comprehensive solution for extensive arch pathology requiring ascending aortic replacement, ensuring long-term durability in anatomically complex disease. In contrast, Type IV HAR represents a less invasive alternative for high-risk patients who may not tolerate sternotomy or cardiopulmonary bypass, thereby expanding therapeutic eligibility in fragile populations (18). This stratified approach reflects a broader shift in contemporary aortic surgery from procedure-centered decision-making toward patient-centered strategy selection (11). By aligning anatomical complexity with surgical tolerance, the framework aims to optimize both early safety and mid-term durability.

While total arch replacement with frozen elephant trunk remains a cornerstone technique for extensive arch disease, focusing exclusively on this subset would overlook the broader clinical challenge of modality selection across heterogeneous presentations. In real-world practice, surgeons are often confronted with patients who differ substantially in anatomical extent and operative risk. Therefore, a comprehensive framework encompassing multiple hybrid configurations may better reflect contemporary decision-making patterns.

## Limitations and clinical implications

This study is a single-center retrospective study with a small sample size (especially only two cases of Type Ⅰ HAR), which may introduce selection bias. Moreover, the heterogeneity of lesion types may limit direct subgroup comparison; however, such heterogeneity reflects real-world clinical practice and underpins the rationale for developing a stratified modality allocation framework. The maximum follow-up duration is 3 years; long-term efficacy (e.g., aortic stability and stent durability over 5 years) needs verification with larger samples and extended follow-up. However, short-to-medium-term results show significant advantages of HAR: compared with traditional open surgery, it causes less trauma and fewer complications (suitable for patients with multiple comorbidities); compared with full endovascular repair, it has lower anatomical requirements (no strict anchoring zone or branch vessel diameter limits) and wider applicability, covering more patients with complex anatomy (19). Because high-risk patients with proximal arch lesions were preferentially treated with conventional elephant trunk repair rather than hybrid techniques, the present cohort does not represent the entire spectrum of arch pathology. This selection strategy may limit the generalizability of our findings and should be considered when interpreting the results. In addition, because treatment allocation was determined according to predefined anatomical and surgical risk criteria rather than randomization, direct comparison between different hybrid configurations may be subject to selection bias. Therefore, the present results should be interpreted as a descriptive evaluation of safety within each subgroup rather than evidence of comparative superiority.

Future clinical practice should focus on two optimizations: (1) cervical approach for Type Ⅳ HAR: explore endoscopic technology to reduce incisions and lower recurrent laryngeal nerve injury risk. (2) CPB duration for Type Ⅱ HAR: attempt “beating-heart anastomosis” to shorten myocardial ischemia time and accelerate postoperative cardiac function recovery. In addition, a long-term follow-up database should be established to systematically record aortic morphology and functional changes at 5 and 10 years postoperatively, providing sufficient evidence for the long-term efficacy of HAR.

## Conclusion

In summary, individualized HAR guided by anatomical and risk-based stratification achieved favorable short- to mid-term outcomes in this cohort. Although larger multicenter studies with longer follow-up are required, the present findings suggest that structured modality selection may represent a rational strategy for managing heterogeneous aortic arch lesions, particularly in patients at elevated operative risk.

## Data Availability

The original contributions presented in the study are included in the article/Supplementary Material, further inquiries can be directed to the corresponding author.
